# Spatiotemporal Metabolome analysis reveals a metabolic network during development of the waxy sorghum landrace ‘Hongyingzi’

**DOI:** 10.3389/fpls.2026.1806648

**Published:** 2026-04-07

**Authors:** Jibin Wang, Qiong Li, Mo Chen, Yuyu Chen, Yanchun Peng, Songxian Yan

**Affiliations:** 1 School of Resources and Environment, Moutai Institute, Renhuai, Guizhou, China; 2 School of Brewing Engineering, Moutai Institute, Renhuai, Guizhou, China; 3 Hubei Academy of Agricultural Sciences, Wuhan, Hubei, China; 4 Guizhou Engineering Research Center for Comprehensive Utilization of Distillers’ Grains, Renhuai, Guizhou, China

**Keywords:** Hongyingzi, key regulatory genes, metabolic network, sorghum, whole life cycle

## Abstract

**Introduction:**

Waxy sorghum is widely utilized in the production of commercial brewing products in China. However, the variations in metabolic profile across its whole life cycle have not been characterized, though several studies have been conducted in certain tissues. This study aims to systematically map the dynamic metabolic landscape and identify the key regulatory nodes across the complete developmental trajectory of a representative waxy sorghum landrace.

**Methods:**

This study systematically analyzed the accumulation patterns of metabolites across different developmental stages of ‘Hongyingzi’, a waxy sorghum landrace. Samples of fifteen tissues were collected at eight key developmental stages.

**Results and discussion:**

Broad metabolomics identified 1,324 metabolites belonging to 12 distinct classes. Tissue-specific metabolic profiling revealed that stems and grains at early developmental stages had high accumulation of phytohormones, whereas the roots contained abundant allelopathicals. Additionally, spikelets and mature grains were enriched with antibacterial alkaloids and a putative nove class of immunostimulatory nucleoside bases, implying that these metabolites are involved in biotic and abiotic stress responses. Integrative analysis of the metabolomic and transcriptomic data resulted in the construction of a metabolic regulation network, which was used to identify the key regulatory genes. For instance, a C-glycosyltransferase gene (*CGT*) associated with high flavonoid accumulation and its co-expressed MYB transcription factor were identified in leaves, while a phospholipase D gene (*PLD*) and an MYB transcription factor related to lipid metabolism were detected in roots. The results provide a systematic profile of the dynamic metabolic changes and tissue-specific regulatory mechanisms throughout the life cycle of waxy sorghum, providing valuable resources and insights for understanding the metabolic basis of its key agronomic traits. The network identified can serve as a foundation for future organ-specific chemical defense studies and targeted crop improvement.

## 1 Introduction

Plant metabolites are the foundation of plant biological phenotypes, and can be utilized for intuitive and effective understanding of the biological processes and their mechanisms. Sorghum (*Sorghum bicolor L*. Moench) is the fifth most produced cereal crop worldwide for the production of food, fiber, bioethanol, fodder, and brewing products (FAOSTAT, https://www.fao.org/faostat/en/#data). In particular, waxy sorghum is widely used in brewing industry due to its unique glutinous texture and high starch content. Tannins in sorghum are polyphenolic compounds with significant impacts on the flavor, color, and stability of the brewing products. They are important branched metabolites derived from the phenylpropanoid and flavonoid biosynthetic pathways, and their content and composition can be precisely regulated for specific applications.

Metabolomics serves as a powerful tool to explore the metabolic responses of plants to biotic and abiotic stresses. Under stress conditions, plants will undergo significant metabolic reprogramming. Analytical techniques such as Gas Chromatography-Mass Spectrometry (GC-MS) and Liquid Chromatography-Mass Spectrometry (LC-MS) allow comprehensive profiling of these metabolic variations. These techniques enable identification of the key metabolites involved in plant defense mechanisms, thereby providing critical insights for crop improvement strategies (Chapman et al., 2019; Piasecka et al., 2019). The establishment of a high-throughput metabolomics platform for sorghum has enabled the application of metabolomics to numerous aspects of research on sorghum biology. For example, it has been used to reveal the relationship between sugar metabolism in sweet sorghum grains and stem development, as well as distinct metabolic changes during internode and seed color development (Li et al., 2019; Zhou et al., 2020). In addition, metabolomics has been applied to study sorghum resistance to disease and pest stresses (Xie et al., 2019).

Rapid advancements in high-throughput transcriptomics and metabolomics have enabled comprehensive analyses combining both omics approaches on a larger scale (Hoffmann et al., 2012). Substantial progress has been achieved in metabolomics research on sorghum, particularly in terms of sugar accumulation, abiotic stress tolerance, and antioxidant, nutritional, and medicinal properties of sorghum. For example, a previous study systematically compared the transcriptomic and metabolomic changes in sugar accumulation at various stages of seed filling in different sorghum varieties. Similarly, transcriptome and metabolome data were combined to study the similarities and differences in the response of sorghum to different stresses (Punia et al., 2021; Simpson et al., 2021) and identify the candidate genes (Akbudak and Filiz, 2019; Ma et al., 2020). However, most previous studies have focused on either the whole plants, specific tissues or specific developmental stages. Many differential metabolites and genes exhibit dynamic changes across different developmental stages within a specific tissue and distinct tissue specificity. As a result, the insights drawn from these analyses may be limited due to this spatiotemporal complexity.

In this study, we investigated the metabolic dynamics across the whole life cycle of the waxy sorghum landrace ‘Hongyingzi’ from Guihzou Province, China with samples of fifteen tissues collected at eight key developmental stages. Through broad-spectrum metabolomics, we quantified the metabolites to delineate their tissue-specific accumulation patterns and temporal dynamics, and integrated the transcriptomic data to construct a regulatory network for identifying the key genes. Particular attention was paid to linking metabolic shifts with developmental transitions and tissue functions. The findings provide a valuable resource for understanding the metabolic basis underlying the agronomic traits in sorghum.

## 2 Materials and methods

### 2.1 Experimental design

The sorghum landrace ‘Hongyingzi’ was cultivated under standard field conditions. To systematically profile the metabolome across the plant life cycle, a total of 45 samples were collected, encompassing eight critical developmental stages from vegetative growth to reproductive maturity. The sampling stages included germinal, three-leaf, booting, flowering, milk stage, pre-dough stage, dough stage and physiological maturity. At each stage, specific tissues were harvested, including plumule, radicle, roots, stems, leaves, spikelets, and grains, to ensure comprehensive coverage of major organs at key physiological transitions. For each tissue type, samples were collected from nine healthy plants grown under identical conditions. To account for biological variation, tissues from every three plants were pooled to form one biological replicate, resulting in a total of three biological replicates per tissue for further analysis. All collected samples were immediately frozen in liquid nitrogen.

### 2.2 Plant materials and growth conditions

The plant material used in this study was the waxy sorghum landrace ‘Hongyingzi’, which is a key raw material for Guizhou Maotai Distillery (Group) Hongyingzi Agricultural Science & Technology Development Co., Ltd. Seeds were obtained from the MouTai Institute farm. The field experiment was conducted during the growing season (April to September) in 2022 at the experimental station in Renhuai city, Guizhou province, China. The experiment was conducted in a randomized complete block design with three replicates. The soil was a yellow loam with a pH of 6.2. Standard local agricultural practices for irrigation, fertilization, and pest control were followed. Tissue samples collected at eight critical developmental stages were immediately frozen in liquid nitrogen and stored at –80 °C.

### 2.3 Metabolomic analysis

Metabolome profiling was generated using a widely targeted metabolome method by Wuhan Metware Biotechnology Co., Ltd (Wuhan, China) (http://www.metware.cn/) (Chen et al., 2013). All samples were freeze-dried by using a lyophilizer (Scientz-100F) and ground by using a grinder (MM 400, Retsch). The tissue powder (50 mg) was weighed and extracted with 1200 μL of -20 °C pre-cooled 70% aqueous methanol, followed by centrifugation for 3min at 12000 rpm. Next, all supernatants were aspirated and filtered through a microporous membrane (SCAA-104, 0.22 μm pore size; Anpel, Shanghai, China, https://www.anpel.com.cn) before the UPLC-MS/MS analysis.

The sample extracts were analyzed using an UPLC-ESI-MS/MS system (UPLC, ExionLC™ AD, https://sciex.com.cn/) and Tandem mass spectrometry system (https://sciex.com.cn/). The analytical conditions were as follows, UPLC: column, Agilent SB-C18 (1.8 µm, 2.1 mm * 100 mm); The mobile phase was consisted of solvent A, pure water with 0.1% formic acid, and solvent B, acetonitrile with 0.1% formic acid. Sample measurements were performed with a gradient program that employed the starting conditions of 95% A, 5% B. Within 9 min, a linear gradient to 5% A, 95% B was programmed, and a composition of 5% A, 95% B was kept for 1 min. Subsequently, a composition of 95% A, 5.0% B was adjusted within 1.1 min and kept for 2.9 min. The flow velocity was set as 0.35 mL per minute; The column oven was set to 40 °C; The injection volume was 2 μL. The effluent was alternatively connected to an ESI-triple quadrupole-linear iontrap (QTRAP)-MS.

The ESI source operation parameters were as follows: source temperature 550 °C; ion spray voltage (IS) 5500 V (positive ion mode)/-4500 V (negative ion mode); ion source gas I (GSI), gas II (GSII), curtain gas (CUR) were set at 50, 60, and 25 psi, respectively; the collision-activated dissociation (CAD) was high. QQQ scans were acquired as multiple reaction monitoring (MRM) experiments with collision gas (nitrogen) set to medium. DP (declustering potential) and CE (collision energy) for individual MRM transitions was done with further DP and CE optimization. A specific set of MRM transitions were monitored for each period according to the metabolites eluted within this period.

Differential expressed metabolites (DEM) were determined by VIP > 1 and fold change (FC) ≥ 2 or ≤ 0.5. The MetaboAnalystR v.1.0.1 was used for OPLS-DA analysis, and extracted the VIP values from this result. The Kyoto Encyclopedia of Genes and Genomes (KEGG) enrichment analysis using ClusterProfiler v.4.6.0 and functional annotation using the KEGG compound database (http://www.kegg.jp/kegg/compound/).

### 2.4 PCA analysis and clustering of metabolometes

Principal component analysis (PCA) analysis and Hierarchical clustering were performed using procomp function and COMPLEXHEATMAP in R software, respectively, to compare the metabolite profiles among different developmental stages and different tissues.

### 2.5 Gene screening and co-expression analysis based on public databases

The molecular mechanisms were investigated by screening candidate structural genes whose tissue-specific high expression patterns, obtained from the sorghum multi-tissue expression profiles in the public PlantNexus database (https://plantnexus.ohio.edu/) (Zhou et al., 2022), matched the accumulation sites of key metabolites. The functions of candidate genes were validated through homology alignment using NCBI BLASTP and phylogenetic analysis. Potential transcriptional regulators were identified by performing Pearson correlation analysis (r > 0.6, p < 0.01) on expression data from the same platform to find genes significantly co-expressed with the candidates in target tissues.

## 3 Results

### 3.1 Metabolic profiling

**Figure 1:**
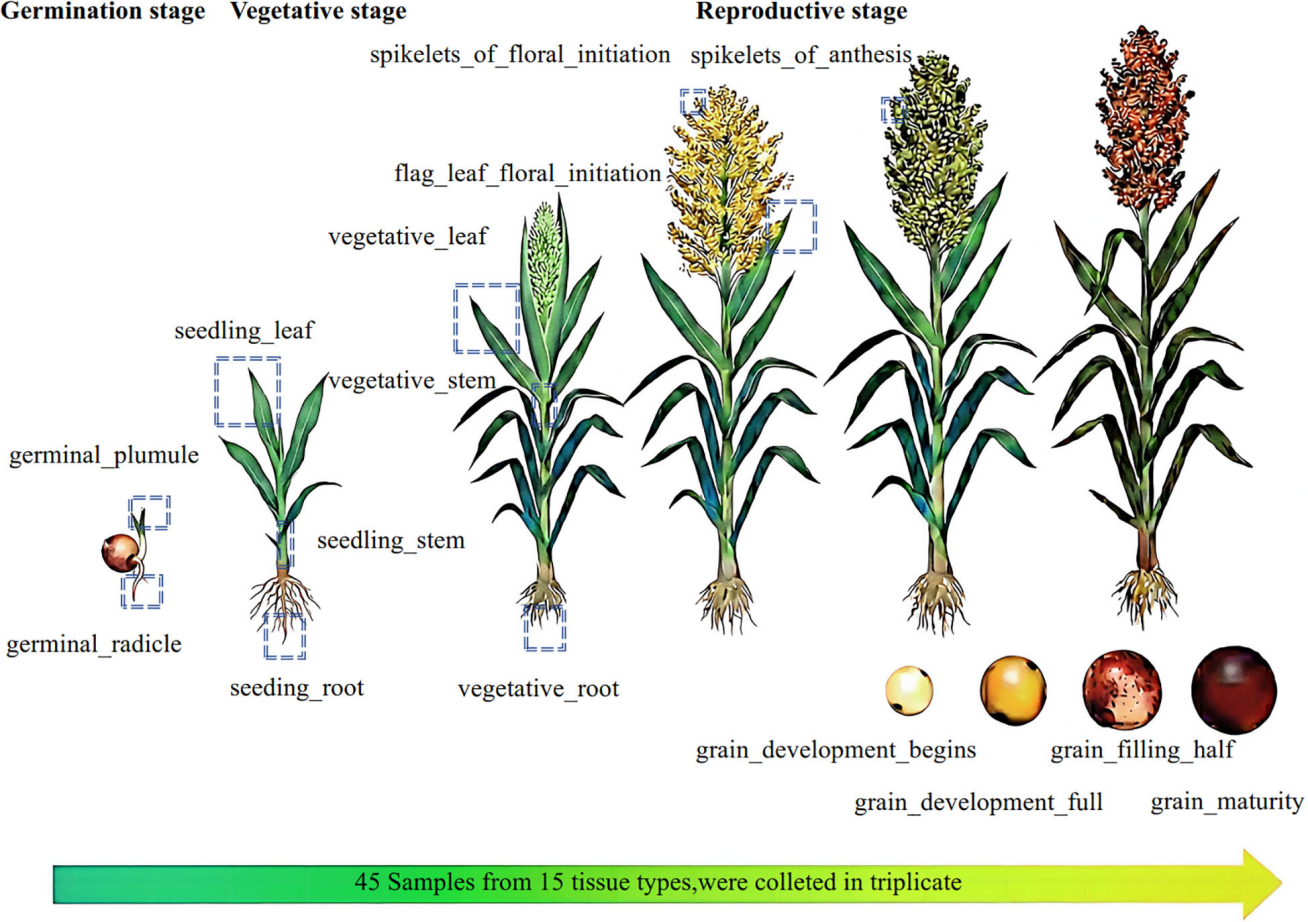
Sorghum growth stages and tissue sampling strategy.

**Figure 2:**
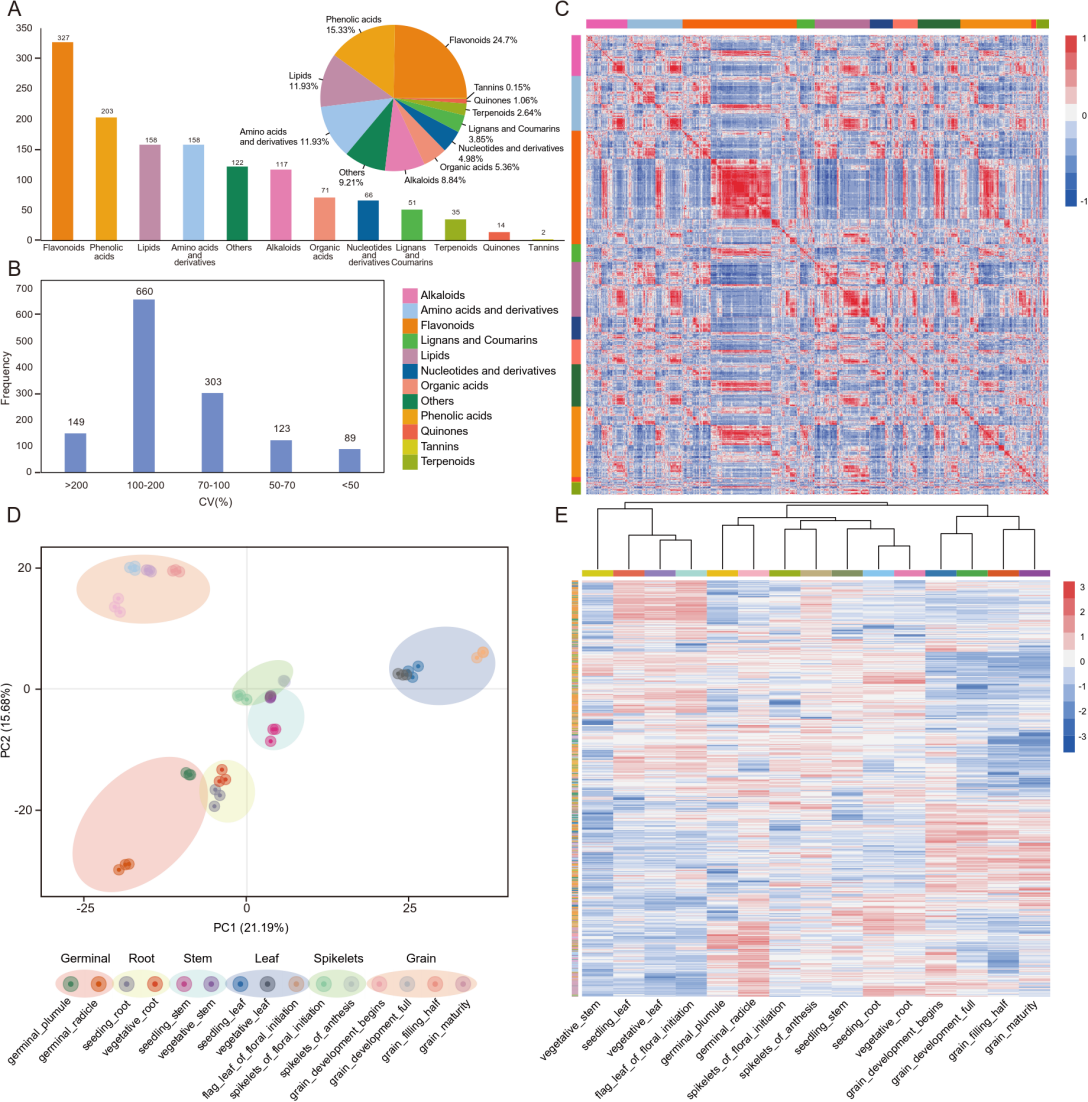
Comprehensive metabolomic analysis of metabolites in sorghum. **(A)** Metabolite classification statistics. **(B)** Distribution of the coefficient of metabolites variation (CV) in metabolite accumulation. **(C)** Pearson correlation analysis of the 1,324 detected metabolites in the relationship between metabolic pathways. **(D)** Principal component analysis (PCA) score plot from 45 sorghum samples. **(E)** Clustering heatmap of metabolite accumulation patterns.

The metabolic profiling involved a total of 45 samples from ‘Hongyingzi’, a waxy sorghum landrace used for Chinese liquor production, covering fifteen tissues of plumules, radicles, roots, stems, leaves, spikelets and grains, and eight developmental stages of germinal, three-leaf, booting, flowering, milk stage, pre-dough stage, dough stage and physiological maturity, with three biological replicates (**Figure 1**; **Supplementary Table S1**). Broad metabolomics profiling identified 1,324 metabolites, which comprised 12 classes, mainly including flavonoids (327, 24.7%), phenolic acids (203, 15.33%), lipids (158, 11.93%), and amino acids and their derivatives (158, 11.93%) (**Figure 2A**; **Supplementary Table S2**). Only 89 (6.7%) metabolites had coefficient of variation (CV) less than 50% (**Figure 2B**), suggesting that further analysis of the metabolome can explain the variations in metabolites during sorghum development.

Pearson correlation analysis was performed on all the detected metabolites to investigate the relationship between metabolic pathways (**Figure 2C**). Two different patterns of correlation were found among the flavonoids. The highly correlated flavonoids were generally derived from the same synthetic pathway with the same catalytic enzyme; while the flavonoids with lower correlations were derived from different synthetic pathways, such as those compounds from the apigenin pathway (flavonoids) and the eriodictyol pathway (flavanones/flavonols), or with a competitive relationship for a common precursor substance naringenin. In addition, there were generally high correlations among lipids, suggesting that these lipids are probably synthesized through related or shared metabolic pathways, possibly involving common precursor molecules or enzymatic steps.

Through metabolome principal component analysis (PCA), it was found that among the 45 sample groups, PCA demonstrated clear metabolic segregation driven by tissue identity. PC1 and PC2 accounted for 36.87% of the total variance, effectively partitioning samples into Grain, Leaf, Stem, Root, and Germinal clusters. Notably, Grain samples exhibited the most significant metabolic divergence along PC1. Within each cluster, samples displayed a clear developmental trajectory. The high consistency among biological replicates confirms robust metabolite profiling. (**Figure 2D**). Further clustering analysis revealed that the germinal stage primarily accumulated alkaloids (pyrrole, indole), organic acids, and lipids; leaves mainly accumulated flavonoids (dihydroflavonoids/flavonols), lignans, and coumarins; spikelets predominantly accumulated flavonols; roots accumulated lipids, terpenoids (diterpenes/triterpenes), and aldehyde compounds; while grains accumulated tannins, nucleotide derivatives, organic acids, chalcones/flavanols, and sphingolipids/LPC (**Figure 2E**).

### 3.2 Specific accumulation pattern

**Figure 3:**
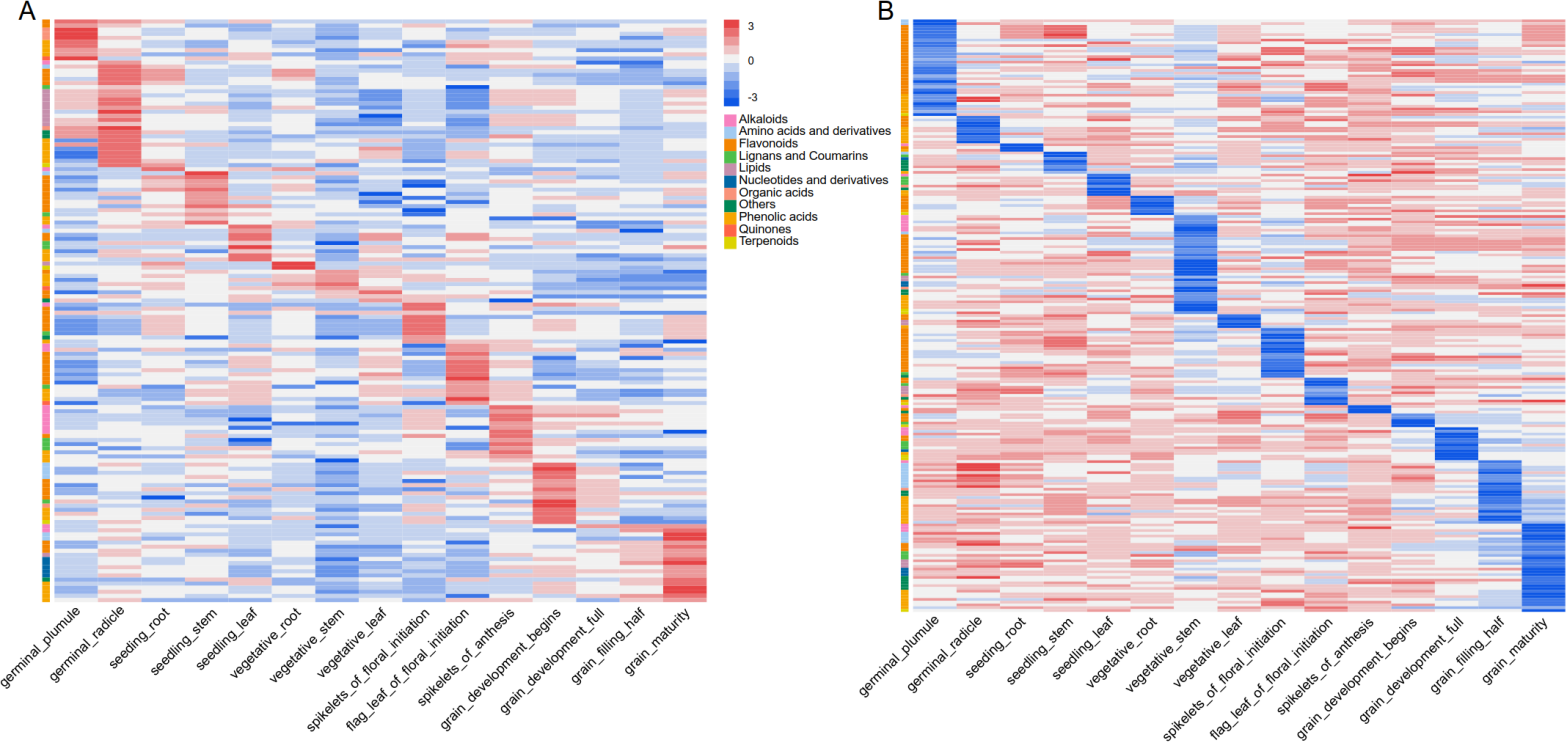
Identification of specific metabolites in developmental stages of sorghum **(A)** Cluster heatmap of high-accumulation specific metabolites across samples. **(B)** Cluster heatmap of low-accumulation specific metabolites across samples.

The accumulation patterns of metabolites at different developmental stages were analyzed by measuring the specifically accumulated metabolites in each sample, and metabolites with FC > 3 or FC < 0.33 relative to other samples were identified as specific high- or low-accumulation metabolites in that sample, respectively. As a result, there were 142 and 215 high- and low-accumulation metabolites, respectively (**Figures 3A, B**; **Supplementary Table 6**). The opposite accumulation patterns of the same metabolite in the same tissue at different stages, as well as in different tissues at the same stage, might reflect the differential flux partitioning between branched metabolic pathway. Opposite accumulation patterns of the same metabolite were also observed in different tissues at the same stage, such as 3-O-galloyl-D-glucose, 1-O-galloyl-β-D-glucose, phenolic acid, antimicrobial alkaloids, which were specifically low in plumule but specifically high in radicle, and may be used to resist biotic stresses in the soil for normal growth and development of the plant (**Supplementary Table 6**) (Khan et al., 2015).

As shown in **Figures 3A, B**, some metabolites exhibited distinct distribution patterns among different tissues and developmental stages. Among vegetative organs, the plumule were specifically enriched with allelochemicals such as malonic acid, suggesting their potential key roles in root defense and microbial interactions (Andama et al., 2020). In contrast, the stems showed specific accumulation of water-soluble compounds such as fructose and sucrose during early growth, which may primarily facilitate rapid plant growth and energy supply (El-Taher et al., 2021). Upon transition to reproductive growth, the mature grain exhibited specific accumulation of diverse antibacterial alkaloids and a putative nove class of immunostimulatory nucleosides. These metabolites together form a crucial chemical barrier against biotic stresses (Kavi Kishor et al., 2022; Wang et al., 2022). Furthermore, the radicle had a high content of malonic acid, suggesting its potential role in regulating cellular oxidative balance against abiotic stress (Hernández-Hernández et al., 2018). These results systematically reflected the spatiotemporal dynamics of metabolites in sorghum during development.

**Figure 4:**
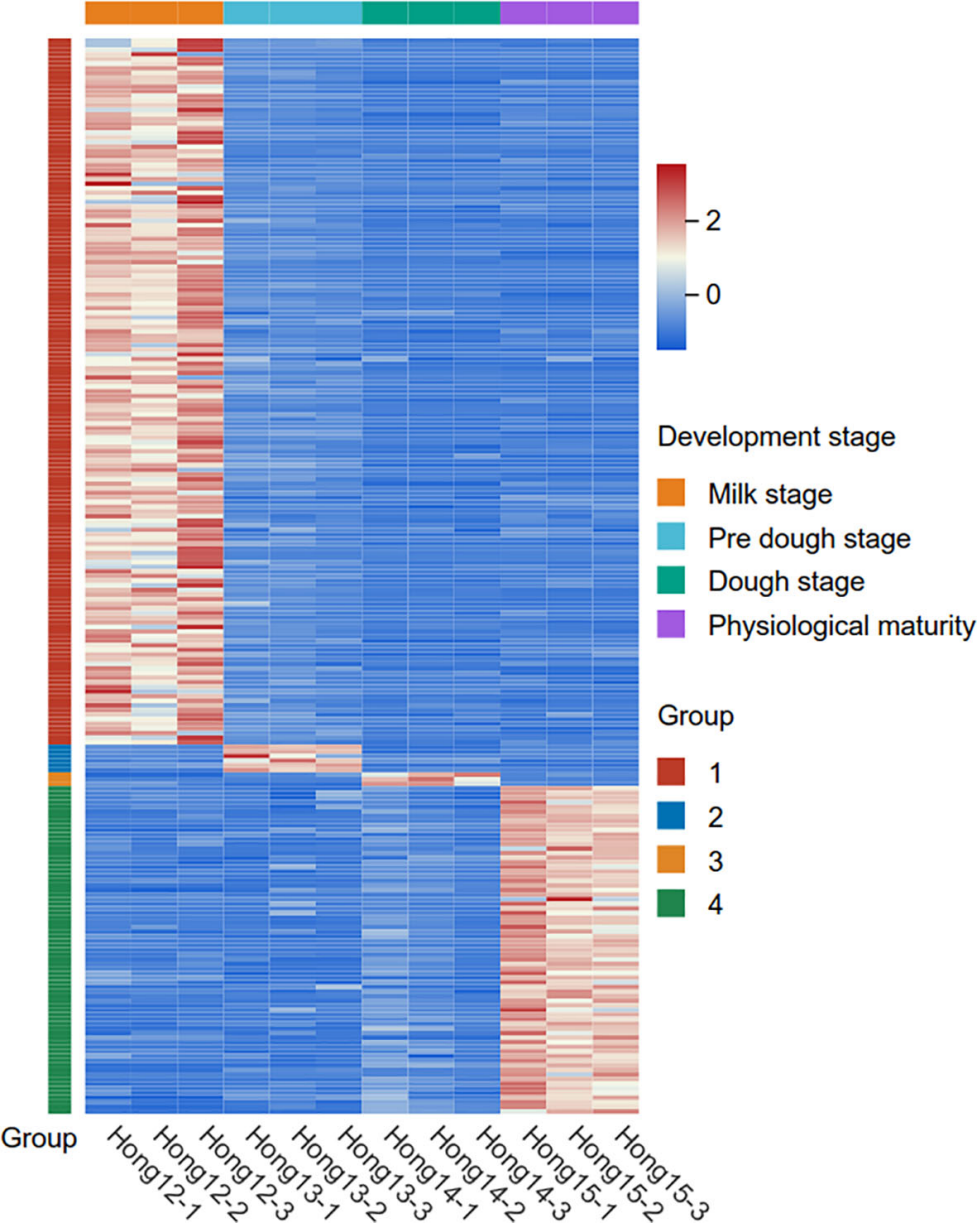
Heatmap of specifically highly accumulated metabolites across four grain developmental stages.

To further elucidate the temporal dynamics of metabolic shifts, we analyzed the accumulation patterns across the four distinct grain developmental stages sample (Hong12, Hong13, Hong14 and Hong15). By comparing each stage against all others, we identified stage-specific metabolites with a FC > 3 or < 0.33 and VIP>1. As a result, we screened a total of 233 stage-specific highly accumulated metabolites. Specifically, the milk stage (Hong12) and physiological maturity (Hong15) exhibited the most distinct metabolic profiles, with 153 and 71 specifically enriched metabolites, respectively. In contrast, fewer stage-specific metabolites were identified during the intermediate phases, with 6 in the pre-dough stage (Hong13) and 3 in the dough stage (Hong14) (**Figure 4**; **Supplementary Table 7**). These results explicitly characterize the dynamic metabolic transitions during grain maturation, highlighting the specialized biochemical activities at each developmental milestone.

### 3.3 Metabolic variations at the grain development stage

**Figure 5:**
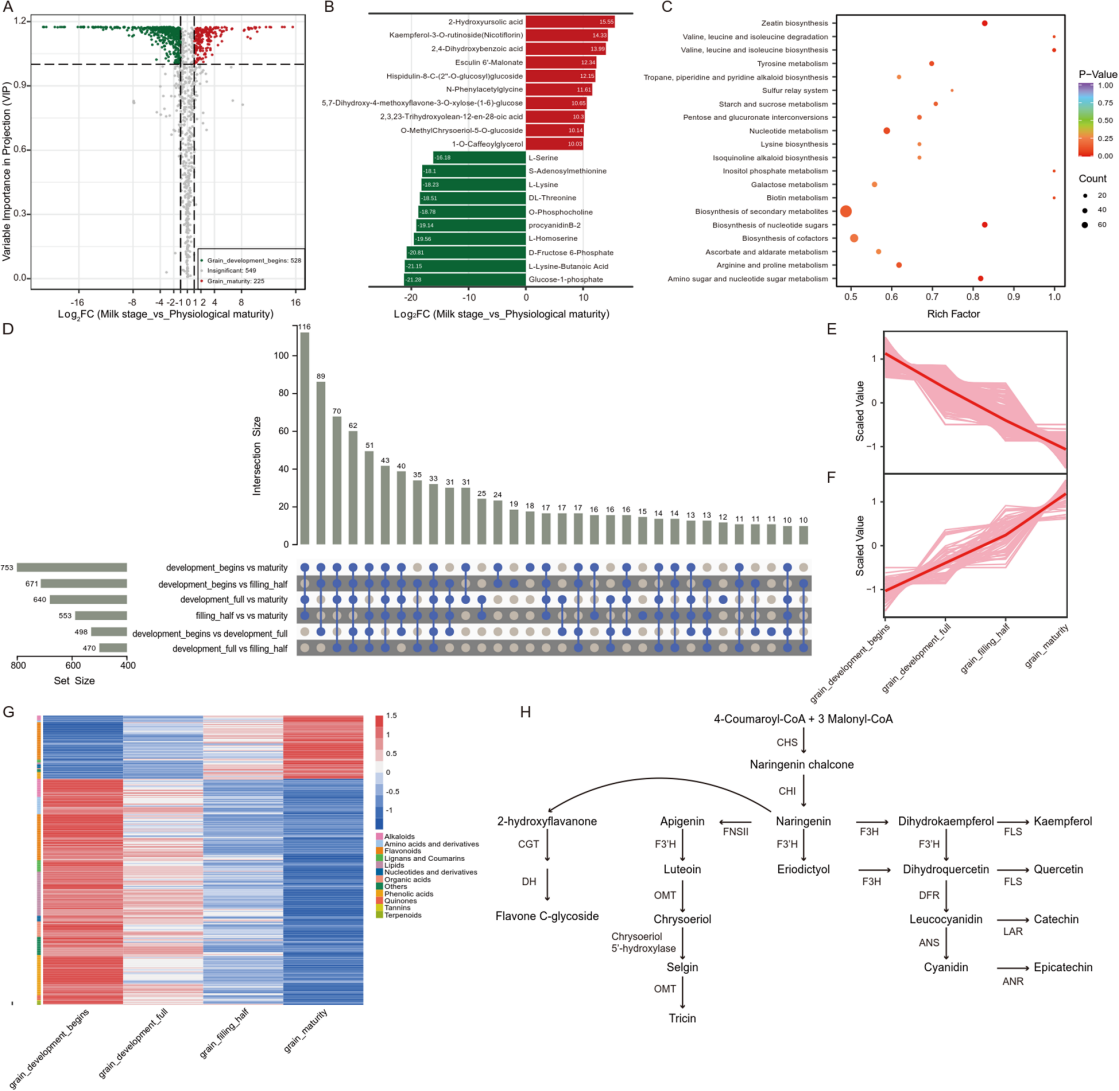
Differential expressed metabolites (DEMs) and their functional pathways during grain development. **(A)** Volcano plot showing significant changes between the beginning of grain development and grain maturity. **(B)** Top 20 most significantly differentially accumulated metabolites (top 10 up-regulated and top 10 down-regulated) comparing grain maturity to early development. **(C)** KEGG pathway enrichment analysis. **(D)** Advanced Venn diagram (UpSet) results for the metabolome data from four grain developmental stage. **(E, F)** Dynamics of screened DEMs across four grain developmental stages. **(G)** Clustering heatmap showing the accumulation patterns of metabolites. **(H)** Schematic representation of the flavonoid biosynthesis pathway.

Grain samples covered four developmental stages and were grouped with pairwise comparison of differential expressed metabolites(DEMs) analysis, resulting in six sets of differential data, which were summarized and de-replicated to generate a total of 1,052 metabolites with differences in at least one comparison group. The smallest difference was found between grain_development_full and grain_filling_half, and the largest difference was detected between grain_development_begins and grain_maturity, with a total of 753 DEMs, among which 225 DEMs were hyperaccumulated at maturity. The metabolites with the highest FC were 2-hydroxyursolic acid (terpenoids), kaempferol-3-O-rutinoside (flavonoids), 2,4-dihydroxybenzoic acid (phenolic acid), 6’-malonylchinocandin (lignans and coumarins), homocarpine-8-C-(2’’-O-glucosyl)glucoside (flavonoids), N-phenylacetylglycine (amino acid and its derivatives), 5,7-dihydroxy-4-methoxyflavone-3-O-xylulose-(1-6)-glucose (flavonoids). At the onset of grain development, 528 metabolites were significantly more abundant, with the highest FC values for glucose-1-phosphate, L-lysine butyrate (amino acid and derivative), D-fructose-6-phosphate, L-homoserine (amino acid and derivative), protoanemonin B-2, O-phosphocholine (alkaloid), DL-threonine, and L-lysine (amino acid and derivative), among others (**Figures 5A, B**; **Supplementary Figure S1**: **Supplementary Table S4**).

The DEMs between milk stage and physiological maturity were identified via KEGG enrichment analysis, revealing significant enrichment in zeatin biosynthesis and nucleotide sugar biosynthesis pathways (**Figure 5C**). The main metabolite involved in the zeatin biosynthetic pathway was cis-zeatin-7-N-glucoside, which showed significantly higher accumulation at developmental onset. Some studies have reported significant positive correlations between zeatin and indicators such as grain filling speed and grain filling rate, suggesting that the accumulation of zeatin at this stage may contribute to subsequent grain filling and maturation (Cui et al., 2020).The biosynthetic pathway of nucleotide sugars involves the accumulation of nucleoside bases, in which adenine can synthesize a large amount of adenosine triphosphate (ATP). Elevated levels of intracellular ATP will generate a feedback inhibitory effect on the glycolysis pathway, thereby decreasing the conversion of pyruvate to lactate and increasing acetyl-coenzyme A conversion, which can provide sufficient substrates for cell membrane fatty acid synthesis (Lian and Zhao, 2015). ATP elevation promotes acyl coenzyme A thioesterase to enhance the conversion of cell membrane saturated fatty acids to unsaturated fatty acids, thereby improving the resistance of the cell membrane (De Marcos Lousa et al., 2013). It suggested that the early metabolic profile is primed for intensive biomass construction and cellular expansion. The metabolites accumulated at developmental onset are mostly for grain filling and maturation, while those accumulated at maturity are mostly related to stress tolerance and plant self-protection. To understand the gradient accumulation trend of DEMs, we analyzed the data from two perspectives. Firstly, we performed an overlap analysis on six groups of DEMs, and statistically constructed the Upset (Wayne) plots. As a result, 33 out of the 1,052 DEMs were significantly different among the six groups of DEMs, and 25 DEMs showed a clear trend of gradient accumulation (**Figure 5D**; **Supplementary Table S3**). With the proceeding of development, only isoprenoid-7-O-(6’’-feruloyl)glucoside (flavonoids) showed a gradually accumulating trend; while the rest of the lipids (glycerol esters and LPE), phenolic acids, and flavonoids (chalcones and epigallocatechin) showed a gradually decreasing trend, which may be stored abundantly as nutrients at developmental onset, and gradually depleted during the metabolic processes along with development.

Then, we further screened the 1,052 DEMs and retained a total of 302 DEMs with gradient accumulation, among which 66 DEMs (such as alkaloids and flavonoids) were accumulated at progressively higher levels, and 236 DEMs (such as flavonoids, lipids, and phenolic acids) at progressively lower levels (**Figures 5E–G**).This metabolic shift supports the biological necessity of protecting the embryo and storage tissues during the final drying and dormant phases. The differential accumulation of flavonoids could be attributed to the fact that those accumulated during maturation were mainly flavonoids in the apigenin pathway, while those with a gradually decreasing trend were mostly flavanones and flavonols in the eriodictyol/quercetin pathway (**Figure 5H**). The vitamin and niacinamide showed a gradually decreasing trend during grain maturation, which is generally in agreement with the results reported in rice studies. These results indicate that the content of vitamin B, which were involved in vitamin B6 metabolism, nicotinate and nicotinamide metabolism, and pantothenate and CoA biosynthesis, gradually decreased during grain maturation, and the decrease was related to the level of cellular and physiological changes at different developmental stages. (**Supplementary Table 8**) (Miraji et al., 2020; Yang et al., 2022).This metabolic shift supports the biological necessity of protecting the embryo and storage tissues during the final drying and dormant phases.

### 3.4 Mechanism for specific accumulation of metabolites in tissues

**Figure 6:**
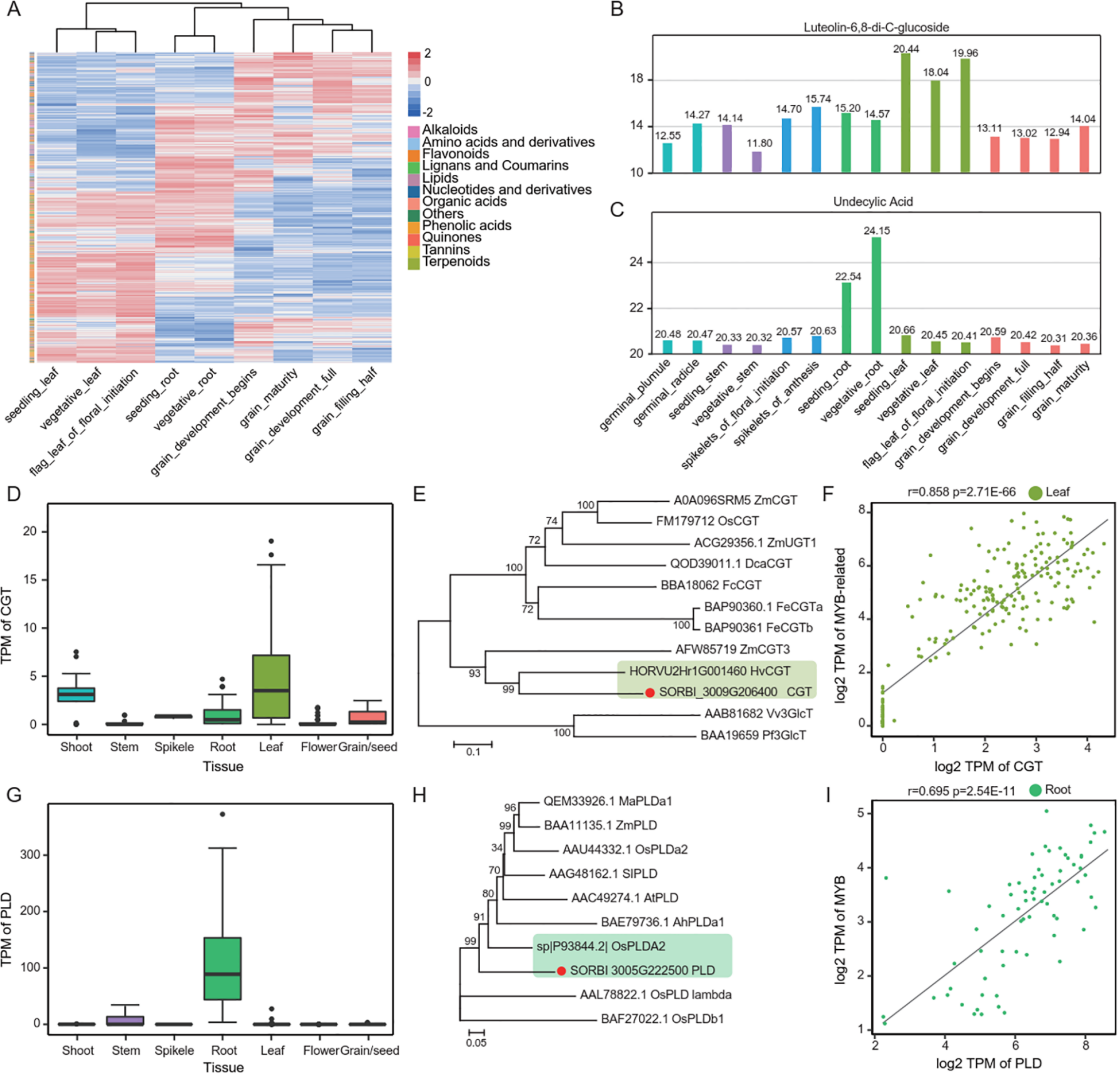
Analysis of metabolite abundance, key gene expression, phylogeny and gene correlation across tissue difference **(A)** Heatmap displaying the relative abundance of various metabolites across different tissues and developmental stages. **(B, C)** Aaccumulation levels of luteolin-6,8-di-C-glucoside **(B)** and undecanoic acid **(C)** in various tissues. **(D, G)** Expression patterns of illustrating the transcripts per million (TPM) distribution of *CGT* **(D)** and *PLD* **(G)** gene across different tissues. **(E, H)** Phylogenetic trees of *CGT* **(E)** and *PLD* **(H)** gene and their homologs in other plant species. **(F, I)** Correlation between the expression levels of *MYB a*nd *CGT* gen*e* in leaves **(F)**, and between *MYB* and *PLD* gene in roots **(I)**.

Clustering analysis revealed that samples from the same tissue at different developmental stages tended to be clustered together, indicating marked tissue-specific metabolite accumulation patterns. Pearson correlation analysis confirmed strong internal correlations within each tissue, including roots (vegetative_root and seedling_root), leaves (seedling_leaf, vegetative_leaf, and flag_leaf_of_floral_initiation), and grains (grain_development_begins, grain_development_full, grain_filling_half, and grain_maturity). Given the high within-group correlations, pairwise comparisons between different tissues were performed to identify DEMs (**Figure 2E**; **Supplementary Figure S2**). A total of 974 metabolites were significantly different in at least one set of comparisons. Statistical analysis revealed the specific accumulation patterns of metabolites in each tissue: lipids and terpenoids were mainly accumulated in the roots; flavonoids were mainly found in the leaves; and tannins, nucleotide derivatives, and other metabolites were mainly present in the grains (**Figures 6A**; **Supplementary Table S5**). Then, the molecular mechanisms were analyzed to screen new genes using the data of gene expression and co-expression network (https://plantnexus.ohio.edu/) (Zhou et al., 2022).

Lignan-6,8-di-C-glucoside, a flavonoid metabolite, showed high accumulation in leaves, which has antioxidant and antimicrobial effects (Tchoumtchoua et al., 2019; Llorent-Martínez et al., 2022) (**Figure 6B**). We screened the *CGT* (C-glycosyltransferase, SORBI_3009G206400) gene with the same tissue expression trend as lignansin-6,8-di-C-glucoside in our database, and the gene showed significantly high expression in the leaves (**Figure 6D**). Protein sequence homology comparison revealed that this *CGT* gene is highly homologous to the reported flavonoid C-glycosyltransferase gene HORVU2Hr1G001460 (Yu et al., 2022), which were clustered in the same branch in the phylogenetic tree (**Figure 6E**). We further screened the transcription factors co-expressed with this *CGT* gene through Pearson correlation analysis, and found that a MYB-related (SORBI_3003G223400) transcription factor was significantly highly correlated with *CGT* in leaf samples (cor = 0.858, p = 2.71E-66) (**Figure 6F**).

Undecanoic acid, a lipid metabolite with antifungal and other effects, was highly accumulated in roots (**Figure 6C**) (Voicu et al., 2004). We then screened the database for the gene *PLD* (SORBI_3005G222500) with the same tissue expression trend as undecanoic acid and significant high expression in roots (**Figure 6G**). Protein sequence homology comparison revealed that *PLD* is highly homologous to OsPLD alpha2, which has been reported to play a role in lipid synthesis in chloroplast lipid metabolism (McGee et al., 2003), and was clustered in the same branch with *PLD* in the phylogenetic tree (**Figure 6H**). We further screened for the transcription factors co-expressed with *PLD* by Pearson correlation analysis and found that a *MYB* (SORBI_3003G053200) transcription factor was significantly highly correlated with the sorghum *PLD* gene in roots (cor = 0.695, p = 2.54E-11) (**Figure 5I**).

To contextualize these candidates within broader plant biology, we identified their orthologs in *Arabidopsis thaliana*. The Sorghum *CGT* gene (SORBI_3009G206400) showed high similarity to Arabidopsis AT5G17050 (UGT78D2), a UDP-glycosyltransferase implicated in flavonoid glycosylation. The Sorghum MYB-related (SORBI_3003G223400) is homologous to AT5G49330 (MYB111/PFG3), a key regulator of flavonoid biosynthesis. Similarly, the PLD-associated MYB (SORBI_3003G053200) shared homology with Arabidopsis lipid metabolism regulators. The existence of functionally characterized orthologs supports the putative roles for the sorghum genes and provides a rationale for future functional validation, such as using corresponding Arabidopsis mutants to establish causal relationships. Subsequent experiments are required to validate the gene-related functions.

## 4 Discussion

This study constructed a comprehensive spatiotemporal metabolomic atlas for the waxy sorghum landrace ‘Hongyingzi’, and characterized its dynamic metabolic landscape across major tissues and developmental stages. The results demonstrated that the sorghum metabolome has undergone precise reprogramming tightly associated with tissue identity and developmental progression, which is a phenomenon increasingly documented in plant systems biology. Principal component analysis revealed pronounced separation of metabolic profiles among tissues, underscoring high degrees of metabolic specialization, which is consistent with the findings in other crops such as rice and maize (Troupin et al., 2016). The distinct accumulation of allelopathicals compounds in sorghum roots highlighted their pivotal role in below-ground ecological interactions, including defense against soil-borne pathogens and modulation of the rhizosphere microbiome. These compounds, often phenolic acids and related derivatives, have been documented to inhibit competing plant species and affect microbial community composition (Kong et al., 2019; Fan et al., 2025). In contrast, the enrichment of antimicrobial alkaloids and a putative nove class of immunostimulatory nucleobases in spikelets and mature grains revealed a sophisticated, organ-specific chemical defense strategy in reproductive tissues. While the exact structure and immune-stimulatory activity of these nucleobases require further identification and bioassay, their specific accumulation patterns strongly suggest their roles in protecting reproductive structures, which aligns with known strategies of chemical defense allocation. This defensive enrichment probably protects developing seeds from infection and predation, reinforcing seed viability (Wang et al., 2022). Furthermore, there was a metabolic shift from growth-promoting, water-soluble compounds at early stages to defense-related, specialized metabolites at maturity stage, which agrees with the ecological principle of growth-defense trade-off, implying the strategic allocation of metabolic resources throughout the life cycle.

A key finding in this study is the close metabolic relationship between the germinal stage and mature grains, indicating a possible metabolic priming effect or early establishment of pathways crucial for grain quality, which is an area meriting further studies. The extensive use of trend and correlation analysis can decipher these complex spatiotemporal patterns, going beyond static snapshots to reveal developmental trajectories and potential functional associations within the metabolic network. Here, the integration of metabolomic and transcriptomic data bridged metabolic accumulation with genetic regulation. The correlation between *CGT* and its co-expressed MYB transcription factor in leaves with high flavonoid accumulation is supported by prior research on flavonoid biosynthesis. Concurrently, the association of *PLD* with an MYB transcription factor in roots offers novel insights into the transcriptional control of lipid metabolism in sorghum (Kumar et al., 2022; Imran et al., 2025). The identification of these candidate genes via co-expression analysis, supplemented by cross-species homology inference, provides a starting point for mechanistic studies. Future work involving genetic manipulation in model plants or sorghum itself is essential to move from correlation to causation and fully delineate these regulatory modules.

In this study, flavonoids, lipids, and phenolic acids were identified as pivotal DEMs throughout the development of sorghum grains. Existing research has suggested that these metabolites exert a direct influence on the flavor profile of fermentation products and indirectly modulate the fermentation process by shaping the composition and functionality of microbial communities. Firstly, flavonoids demonstrate considerable biological activities during fermentation. It has been demonstrated that flavonoids can modulate starch digestion through the formation of non-covalent complexes with starch molecules. *In vitro* fermentation experiments have shown that flavonoids facilitate the proliferation of beneficial bacteria such as Lactobacillus and Bifidobacterium, while concurrently inhibiting the growth of *Escherichia coli* (Pan et al., 2023). This finding implies that flavonoids not only have a direct impact on the flavor of fermentation products, but also indirectly affect the fermentation process by regulating the composition and metabolic activity of microbial communities. Secondly, lipids also play a crucial role in fermentation. Research has demonstrated that during the fermentation of nuts with varying particle sizes, such as almonds and macadamia nuts, the presence of lipids and the particle size significantly influence the dynamics in microbial communities (Cai et al., 2024). Lipid degradation is intricately linked to microbial adhesion and proliferation, suggesting that this interaction may be essential for lipid breakdown, thereby influencing the flavor profile of fermentation products. Phenolic acids play a crucial role during fermentation, as research has demonstrated their interactions with microbial metabolic processes, thereby influencing the flavor and quality of the final products. Specifically, during fermentation, the metabolic pathways of phenolic acids are closely associated with lipid and amino acid metabolism, among others. Alterations in these pathways have a direct effect on the flavor characteristics of fermentation products (Bermudez et al., 2024).

## 5 Conclusions

Our results demonstrated that the waxy sorghum landrace ‘Hongyingzi’ exhibits distinct metabolic defense strategies across various tissues. Notably, elevated levels of allelochemicals were detected in the roots, indicating that this variety possesses significant habitat competitive advantages during early growth. Concurrently, the enrichment of antibacterial alkaloids and immunomodulatory nucleosides in spikelets and mature grains constitutes a “chemical barrier” during late seed development. These pivotal DEMs are essential for resisting pathogen invasion and have potential effects on the flavor during the brewing fermentation. This discovery not only elucidates the resource allocation strategies developed by sorghum through evolution but also provides definitive metabolic markers for the screening of highly resistant breeding materials (Xie et al., 2019; Ma et al., 2020).

## Data Availability

The original contributions presented in the study are included in the article/**Supplementary Material**. Further inquiries can be directed to the corresponding author.

## Supplementary material

Supplementary Figure 1 Heatmap visualization of metabolite accumulation patterns during sorghum grain development.

Supplementary Figure 2 Pearson correlation analysis on metabolite accumulation exhibiting significant tissue specificity and developmental stage specificity.
