# Supplementary figures and images for: Spatiotemporal Metabolome analysis reveals a metabolic network during development of the waxy sorghum landrace ‘Hongyingzi’
Source: Front Plant Sci. 2026 Apr 7;17:1806648. doi: 10.3389/fpls.2026.1806648 (PMC13095745; doi:10.3389/fpls.2026.1806648)

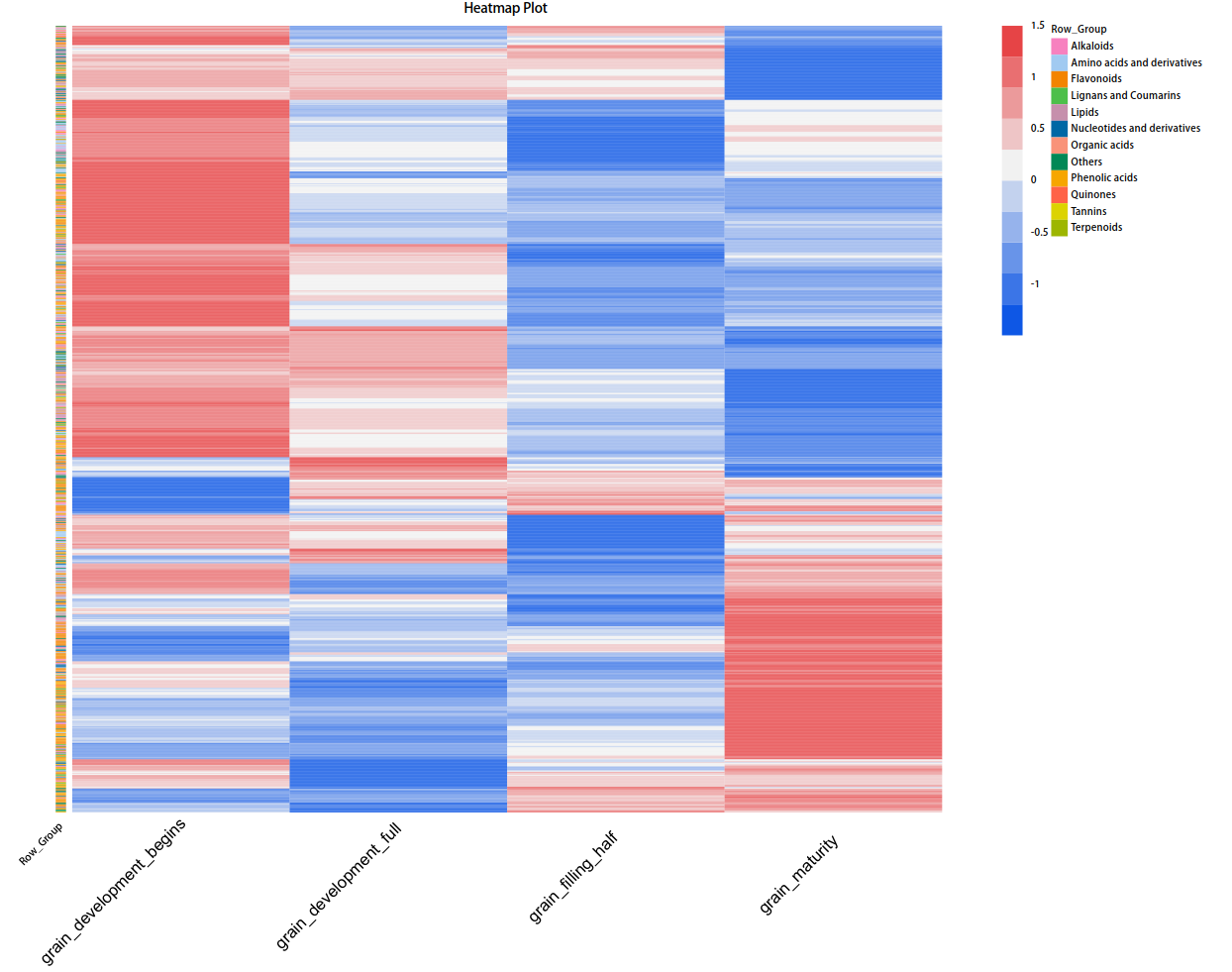

Supplement: Supplementary Figure 1 — Heatmap visualization of metabolite accumulation patterns during sorghum grain development. [file Image1.tif]

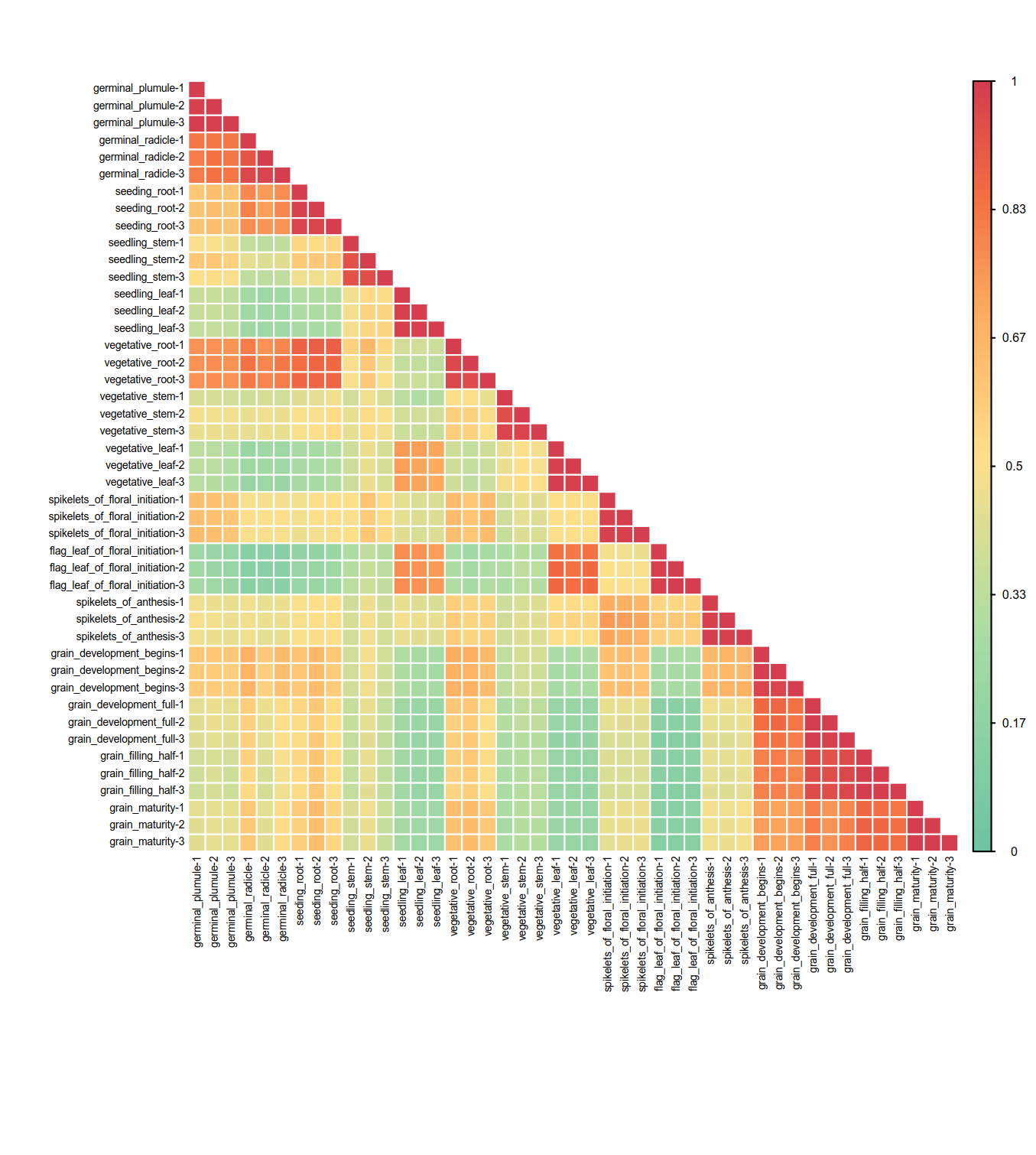

Supplement: Supplementary Figure 2 — Pearson correlation analysis on metabolite accumulation exhibiting significant tissue specificity and developmental stage specificity. [file Image2.tif]
